# Insight from Women on the Lived Experience of Their Partners’ Referrals to Men's Behavior Change Programs

**DOI:** 10.1177/10778012251338487

**Published:** 2025-05-08

**Authors:** Lauren Zeuschner, Marg Camilleri

**Affiliations:** 1 Institute of Education, Arts and Community, 1458 Federation University, Ballarat, VIC, Australia

**Keywords:** men's behaviour change, women's experiences, family violence, referral, feminist interpretative phenomenological analysis

## Abstract

Within the state of Victoria (Australia), male perpetrators of family violence are routinely referred to Men's Behaviour Change Programs (MBCP). This place based-based study included nine in-depth interviews with women from a regional city in Victoria, about their lived experiences of their partners’ referral to an MBCP. The women's shared narratives were explored via Feminist Interpretative Phenomenological Analysis. This analysis exposed the referral period as a time of pivotal assessment for the women – a time characterized by hope, blame, judgement, and indignation. This study emphasizes the need for community services to take into account the far-reaching implications an MBCP referral has for everyone it is intended to benefit.

## Introduction

One in four Australian women experience at least one incident of family violence (FV) perpetrated by a male partner (current or former) in their lifetime (Australian Institute of Health and Welfare, 2024). The Victorian State Government recognizes FV as a product of gender inequality (State Government of Victoria, 2021), an inequality which has, in part, been sustained by a patriarchal social structure whereby violence and oppression are utilized by men to uphold a position of power over women (Grosser & Tyler, 2022). Silencing the voices of some while privileging the voices of others is one such example, a reality which has extended to research into Men's Behaviour Change Programs (MBCPs).

In 2015–2016, the Australian Royal Commission Victoria (RCFV) held a 13-month inquiry into FV and concluded that to prevent FV, and improve the related service delivery, it was crucial that the voices of victim survivors be heard (RCFV, 2016). Prior to this study, meaningful consultation with women regarding their experiences of the MBCP referral made for their partners had not occurred—their experience-based knowledge had effectively been excluded from the conversation.

Consequently, assumptions underpinning MBCP referrals were allowed to continue without genuine input from victim survivors. For example, within Australia MBCPs are championed as a pro-feminist approach to perpetrator intervention, designed to challenge societal beliefs fostering gender inequality (Chung et al., 2020). However, as demonstrated by numerous international researchers, the outcomes of these programs do not offer consistent or verifiable evidence of their effectiveness (Gray et al., 2016; McGinn et al., 2021). Furthermore, while police in the State of Victoria actively refer perpetrators to MBCPs, most do not lead to program completion, or notably, even program attendance (Chung et al., 2020).

### MBCP Literature: Overview and Response

This study began with a systematic review of the existing MBCP literature available at that time and found that while many studies had been conducted around men's experiences, comparatively little attention had been paid to women whom the referral is intended to benefit. Furthermore, the review revealed that a comprehensive understanding of the potential implications for women of having a partner referred was lacking. For a full account of the systematic search of MBCP literature that took place, see Zeuschner (2022). In summary, 11 studies (out of 1839 results), were found to have included victim survivors. Eight of these were mixed methods studies focused on evaluating program outcomes (see Gray et al., 2016; Hossain et al., 2014; McConnell & Taylor, 2016; Milner & Singleton, 2008; Stanley et al., 2012), or program methods (see Alexander et al., 2010; Musser et al., 2008; Richards et al., 2004). All eight studies indicated that the reason victim survivors were included was to verify information obtained from MBCP participants. Two of the 11 studies used quantitative methods to explore perpetrator “readiness to change” (see Eckhardt et al., 2008; Simmons et al., 2008), and the eleventh study (see Morran, 2013) took a qualitative approach to ascertain “what processes and experiences might be involved” for participants who could now be described as “nonviolent” (p. 308). As with the eight mixed methods studies, women were only included in these three remaining studies to verify information obtained from MBCP participants.

In a response contrary to placing women on the periphery of MBCP literature, and in line with the sentiments of the FV reform underway at the time, it was determined that much could be gained from focused research into women's experiences of their partners’ referral to an MBCP. This article reports on the findings of the work that followed, drawing attention to the voices of women as victim survivors of FV, and elevating their insights in a crowded space where men's voices have dominated. At the time of writing, the Federal Government is considering the implementation of a National Plan to End Violence Against Women (National Plan) (Commonwealth of Australia, 2022). MBCPs are included in the National Plan; however, with a greater focus on measuring the effectiveness of programs. Also included is an added emphasis to “…ensure men are prepared and able to engage with these programs and that programs adhere to minimum standards” (p. 84). At this stage it is unclear if measuring the effectiveness of MBCPs will include the experiences of their partners (current or former), or indeed whether services for women will also be focused on supporting them during the period of their male partners involvement in the program.

## Methodology

A feminist-aligned methodology was sought for this study, so that structural factors surrounding and shaping FV would underpin and inform all aspects of the research process. This resulted in the adoption of an emerging constructivist approach titled Feminist Interpretative Phenomenological Analysis (feminist IPA). Unlike positivist-informed research, which typically explores the quantitative manifestations of an issue (Kaboub, 2008), research grounded in constructivism recognizes that the intricacies of lived experience cannot be revealed through a narrow focus on observable data alone. A hermeneutic phenomenological epistemology furthers this position by embracing the idea that while individual experiences of social events may be similar, they are never identical (Bowden & Mummery, 2009). In alignment with these grounding positions, both feminism and IPA “attempt to find modes of discourse, voice, and expression that can reveal felt meaning that goes beyond the prevailing paradigm of logic, cognition, prediction, and control” (van Manen, 2016, p. xvii).

The term “feminist IPA” has been used to establish the research first and foremost as a feminist study seeking to uphold the key tenets of feminist social research, by challenging and expanding male-centric sources of knowledge, while creating an opportunity for women to advance social scientific wisdom (Burgess-Proctor, 2015; Gringeri et al., 2010). Given that prior to this study, meaningful consultation with women regarding their experiences of the MBCP referral made for their partners had not occurred—this feminist approach was considered essential for expanding current understandings. The term also demonstrates the role of IPA in both the design and implementation of the research. This qualitative approach involves inviting and listening to someone's narrative account of their experience, before then interpreting the meaning they have made of it for the purpose of expanding understanding (Alase, 2017; Bynum & Varpio, 2018; Smith & Nizza, 2021). As a result of taking a feminist approach to IPA, the complexities and nuances of the women's lived experiences were safely shared, and the wider impact of structural factors were highlighted and acknowledged. Ethics approval for this research was obtained from Federation University Australia (no. A18-010).

### Recruitment

The recruitment approach was designed to affirm women's lived experiences as valuable and needing to be heard, while inviting women to participate. In addition, the strategies employed aimed to facilitate the participation of women who had, and had not, received support from a community service agency. For example, staff from collaborating agencies disseminated information about the research to all women attending their service, regardless of why they were there that day (e.g., they may have been visiting the doctor). A sample of varied community spaces (e.g., community centers; churches; universities; etc.) also displayed the advertisement on the backs of their toilet stall doors.

The primary inclusion criterion required that the individual had experienced their partner being referred to an MBCP. As the foremost aim of the study was to hear and understand the meaning women made of the referral experience, whether the referral had or had not eventuated into their partners engagement with an MBCP was not a limitation to the women's participation in the study.

Additional criteria for participation included women living in the nominated regional city of Victoria who:

- had experienced FV;
- had a partner who was referred to an MBCP within five years of the recruitment period;
- and had children in their care during the referral period.

Although it was acknowledged that some individuals are more likely to experience FV than others; for example, Australia's First Nations women and women with a disability (Women with Disabilities Victoria, 2023), representatives from different population groups were not targeted. Additionally, this study focused on female partners of heterosexual men due to the heteronormative nature of MBCPs in operation at that time in the research location.

The design of the recruitment process aimed to facilitate self-selection into the study, whereby, after being made aware of the study the women would contact the researcher themselves. However, through discussion with collaborating agencies it became apparent that some women who wanted to participate did not have the resources to allow them to make contact (e.g., they did not have access to the internet, they had limited phone credit, etc.). An ethics amendment allowing a third party (e.g., a case worker) to pass on a woman's contact details if they requested this and provided safety information such as safe times to call, was subsequently submitted and approved.

### Introducing the Women Who Took Part

Several women self-selected and contacted the researcher to discuss the study and their possible participation. Subsequently, the experiences of nine women were shared within a qualitative semistructured interview context. Each woman chose a name to be used within the research, as follows: Angeline, Clara, Erin, Fiona, Jane, Janet, Meera, Paige, and Rose. Some also chose names for their partners, while others preferred for them to be referred to as “partner” or “husband.” The women ranged in age from 33 to 51 years, and all forms of FV were represented across their experiences. At the time of their interviews four women were still residing with the perpetrator: two were still married and two had separated relationally. The remaining five women had separated permanently (both physically and relationally). The length of time they had been in a relationship with the perpetrator who received the MBCP referral ranged from between two and 20 years. One of the women identified as “Aboriginal”; one noted that they had migrated to Australia from Asia as an adult; and the remaining seven identified as “Australian”.

The women expressed a range of reasons for requesting to take part in this study. For example, some like Angeline viewed it as an opportunity to have a “voice.” This was something she (and others) not only wanted but also believed was necessary to increase the effectiveness of MBCPs:

When I saw the poster [ ] I thought, “Yeah, I do want to share my experience because in my experience it [the MBCP] wasn’t very effective”, and I thought, “Has this happened to other people where, their partners have just gone along once and come away thinking it was rubbish that they didn't need to go?” And how many people just don't even go? I was interested to know, but, yeah, I just thought I want to say what happened because it felt really ineffective.

Angeline went on to clarify what she meant by “ineffective”:

I thought no one's ever gonna know my side of the story, it's just what he says and he doesn't think he should be there, so how is that going to come out sounding through him? Um, so, yeah, I just felt, yeah, there was no voice, no way to voice my version of events, so, yeah, I felt it was very ineffective.

Others, like Jane, talked about participation as a way of escaping the destructive consequences they connected with remaining silent:

I refuse to keep my mouth shut because I know that isn’t going to solve any problems, either, and that's why I said, “It's healthy for me to vent, it's healthy for me to talk about it, cos otherwise I’ll end up with rage and people turn to substance abuse because they can’t face things”, and I’ve done all those paths, previously, which I knew they’d lead me nowhere but a lot more mess.

The women's reasons for taking part expressed a strong desire to be heard coupled with an understanding that their lived experiences were a necessary part of the story.

### Analyzing the Women's Narratives

The analysis was carried out primarily by the lead researcher, with support by another for the purpose of validity checking. The process systematically progressed through an inductive and iterative cycle of stages as suggested by Smith et al. (2009), and therefore considered the specifics within each woman's experience, before moving to define broader generalizations across the cases.

The first stage involved immersion in the narrative. This began with reading and listening to an interview recording to re-engage with the timing, pace, and emotion of the woman's experience and meaning making, before working through the transcript formulating three different types of exploratory comments: descriptive, linguistic, and conceptual. The second stage in the analytical process involved transforming the researchers’ exploratory comments into emergent themes. In the third stage, emergent themes from right across the narrative that were interpreted as being conceptually similar, were grouped together and assigned a descriptive label. Table 1 is an example of stages one and two, taken from the analysis of Meera's narrative.

**Table 1.** Exploratory Comments and Themes

3. Emergent themes	1. Original transcript	2. Exploratory comments Descriptive = normal text Linguistic = italics Conceptual = underlined
Self-assertion Help seeking Receiving support Empowerment through consensus	One day we had like, I can’t remember what thing it was but wasn’t like something big but we had an argument R: You and your partner? P: Me and my partner. And then my father in-law just said to my partner “oh you don’t know how to control your wife, if that would be my wife I would have slapped her” so he [*perpetrator*] tried to slap me and but then I was like “don’t you dare” and he just stopped, but because that happened in front of my in-laws, and I was just questioning, although he didn’t hit me but it wasn’t like just bringing that hand here and stopping [*acts out a hand coming in to slap her face*] and we had like similar kind of incident twice, or thrice. Once I remember there was, because we had a bit of argument so I had to call my parents because “this is just going beyond my limit, I can’t take it, so can you please come”. So same, similar kind of thing happened there as well but again I was lucky at that time my husband didn’t slap me and then my father for the very first time he jumped in between us and he just said “I hadn’t done this, since I remember myself, and in my family women are like really equal and we respect them, and if you’re gonna treat my daughter like this, I won’t let her, you know bare that” and then my husband, like he's a good person otherwise he, he said “ok I’m sorry” and things started going back on track.	Father-in-law encourages son to slap M. through use of shame. “*you don’t know how…”* M. asserts self / right not to be hit. “*don’t you dare”*. Threat of assault. M. calls parents for help. “*I had to”* – *expressing felt need for external support*. M's father intervenes and asserts a different perspective re: how women are viewed and treated: “*Women are like really like equal and we respect them.”* Consensus between M and her parents protects M. Husband's behaviour confirms for a time M's belief that he is a “ *good person”.*

Stages one to three of the analysis were completed for each case in turn. Once all the women's narratives had been individually analyzed in this way, the final stage commenced. This involved conducting a cross-case analysis, where themes that had not been identified amongst at least two-thirds of the cases were excluded, and descriptive labels relevant to all the women were chosen (Table 2 provides an abbreviated example of this process). The final superordinate labels were taken directly from the women's transcripts and were chosen for their ability to encapsulate the essence and importance of the subordinate themes contained within. The final stage also involved writing a narrative account of the study.

**Table 2.** Cross Case Analysis

	Emergent themes: Angeline	Emergent themes: Erin	Cross case analysis
**Superordinate theme**	The impact of denial	Responsibility	“I was always the big bad wolf”
**Subordinate themes**	Denial	Denial	1. Facing perpetrator resistance
	Anticipating denial of responsibility	x	Theme excluded
	Minimising	x	Reclassified as an act of ‘denial’ and included under: 1. Facing perpetrator resistance
	MBCP group participants support perpetrators denial of FV	Perpetrators justification through ‘Othering’	1. Facing perpetrator resistance
	Assuming and resisting shared responsibility	Assuming and resisting shared responsibility	2. Assuming and resisting shared responsibility
	x	Guilt	Reclassified as a component of: 2. Assuming and resisting shared responsibility
	x	Being held accountable by others	Theme included as it was significant for 8/9 participants: 3. Feeling blamed.

## Findings

The following three superordinate (major) themes and nine related sub-themes, highlight the women's shared experiences. To uphold the idiographic nature of IPA—and the feminist position that not all women are the same—degrees of difference as captured in the nuanced perspectives within these themes, have also been included.

### Theme 1: There Can Be a Rainbow, Can’t There?

The first major theme within this study encapsulates the progression each woman made through two interrelated states (presented here as sub-themes): (a) seeking help; and (b) experiencing heightened thoughts of change. While only one woman explicitly expressed the sentiment “There can be a rainbow, can’t there?” the question and corresponding hope of experiencing change and a new beginning, was conveyed throughout the narratives of all nine women.

#### Seeking help

For each of the women who took part in this study, the referral was brought on when they sought help (e.g., from family, colleagues, community services and/or the police). The women’s experiences of FV did not end at this point; however, their actions did result in an MBCP referral being made for their male partner. Remarkably, the women sought help even though previous attempts had not yielded support. For example, Paige recalled attempting to gain assistance from the police earlier in her relationship:

You know it was going on and it got to the time, the time where he was literally punching the older boy, and he WOULD NOT get out of the house and I had been down to the police and I’d tried to get an IVO [intervention order] on him but they kept saying we've got no proof, “Got no proof” so because I have no proof therefore it was my word against his [the perpetrator]. And they wouldn't put one on him.

It wasn’t until a few years later when (while still in the same relationship) Paige phoned the police as she and her three-year-old child were being physically assaulted by her partner, that they attended her home and escorted him from her property. At that point he was referred to an MBCP.

Seeking assistance amidst an escalation of fear was common for the women. For example, Erin's emergency call described below expressed her immediate need for support as well as a belief that assistance from an external party was required for the violence to stop:

So I was actually propped up in bed and he just flew across the bed and punched me in the head and knocked me out of bed. He perforated my eardrum, ah, and I had a swollen black eye, a cut and things, and I screamed out and my eldest, they came to the bedroom door and I said “Call triple zero”. This wasn’t an isolated event, like there had been, like many times I had been choked and things like that. It wasn't like “My God that's not like you”, but I was really petrified.

Erin's partner was subsequently taken into custody for the night and referred to an MBCP.

#### Experiencing heightened hopes of change

During the early stages of the referral period, the women experienced a compelling “need to know,” regarding whether the family dynamic they hoped for was something they would ever experience with their partner. For example, Fiona recalled:

I was just thinking there can be a rainbow, can’t there? — I thought if it [the MBCP] can help, this was when he sort of had me bluffed, if it's going to work go for it because the explosions were too big and if he could control himself and think of what he says, pull his head in, if it can work then we CAN be a family. I was hoping.

Consequently, all the women appear to have experienced an intense state where their thoughts of change were heightened. For example, Janet noted:

Oh I thought he would [change]! I honestly thought not for ME, I didn’t think he would change for me but I thought he would change for the girls, really thought, that he would make an effort, a conscious effort; he’d been given this opportunity, to show that he can be a better person, that he would learn skills, and understand and better be able to, to I don’t know, manage himself.

The women had these thoughts despite no prior evidence to suggest their partners were willing or capable of change, which Janet's continuing narrative expresses:

… when we were together and I used to say to him, “We need to go and get help; we need to go and talk to someone”, he would say, “No.” He would yell in my face and tell me to “eff off” and “Mind my own business” and that he didn’t have a problem I was the problem. So he would never go to anything like that; he would never think that anything like that was for him.

It would appear that the heightened thoughts of change experienced by the women, were fueled by hope and a sense of trust in the potential influence of the MBCP. This aspect can be seen in the following extract from Paige's narrative:

It was supposed to be really good, you know, the be all and end all of changing everything for the men and all this sort of stuff—I thought he might take things in and I thought things would change. I thought he would understand the cycle of violence and you know, understanding the cycle might get him to change something in his head.

These heightened thoughts were a powerful motivator for the women to stay living with/in relationship with their partner and/or to support his access to their shared children. When Rose reflected on this aspect of the referral experience, she described it as a “waste of time”:

I’ve wasted a year, on hoping and praying and wishing that things are gonna be different, and we’ve worked on things together like you know, I’ve allowed him access to my kids … so at the end of the day I could have moved on a lot sooner, you know what I mean? Like I could have not given him that chance, and you know, at the end of the day if I haven’t of given him that chance I wouldn’t have my baby, yes I understand that, but the men's behavioural change program is big noted so much, like it's oh you know, it's, “It's a great way for the men to realise what they’ve done and move on.” And, you know, that sort of stuff, and it doesn’t do that.

Roses’ experience contrasted with the messages she had received from others (such as community service workers, friends, and the media) regarding the outcomes she could expect—messages that had fueled her sense of hope. As a result, Rose's narrative seems to suggest that if she had understood the realities around perpetrator change and program outcomes, she may have left the relationship earlier.

### Theme 2: “I was Always the Big Bad Wolf”

Being referred to an MBCP effectively identifies the male as a perpetrator of FV; despite this, most of the women experienced being told by numerous parties that they were responsible for the referral and any associated service involvement. This major theme brings together concepts related to “responsibility,” which are presented as the following three sub-themes: (a) feeling blamed; (b) facing perpetrator resistance; and (c) assuming and resisting shared responsibility. While only one woman explicitly stated, “I was always the big bad wolf”, the implications of feeling blamed throughout the referral period were communicated by all the women in various ways.

#### Feeling blamed

Throughout the referral period the women found that many people; including their partners, family members, friends, along with service staff, were primarily focused on her act of phoning the police (or involving services), rather than on her partners violent behaviors. This theme therefore speaks to the experience of what is commonly referred to as “victim blaming.” For example, Meera recalled the following:

My brother wasn’t that supportive he just said: “You are just ruining your marriage because now you have involved the police, so whatever happens to you that is your consequence because you chose to do that.”

Similarly, Jane spoke about the messaging she received from her in-laws:

…they would ring me “He's really upset, he's really crying” and so, I had them all leaning on me to you know “You need to, He is really sorry”— it was always about making him feel better, it didn’t matter about my feelings, or what my kids were experiencing it was about, making him feel better, and him being ok. So I was pushed to be made to feel bad every time.

Being viewed in this way not only reduced the women's support systems, it also hindered many of them from developing or trusting their own perspectives.

#### Facing perpetrator resistance

As the women reflected on life pre-referral, it became apparent that denying responsibility had been a consistent feature of the FV their partners perpetrated. This resistance to accepting responsibility for their behaviors and to being identified as a perpetrator, continued into the referral period. From the women’s perspectives this resistance was sustained through their involvement with the MBCP in two different ways. Some women described a cognitive process that has been interpreted as “Othering,” whereby, their partners positioned themselves in contrast to the other perpetrators/participants. Erin called this “tactic number 33”:

You know if you punch someone in the head well that's nothing because someone else broke someone's leg and that's far worse. And if someone has never been physically violent, well they’ve never been PHYSICALLY violent, these other people have been physically violent, it doesn't matter what level you're on, there's always someone worse, and that's how they are justifying themselves. Now I see that that's just sort of you know, tactic number 33 or whatever [laughs] but, yeah, cos he would say, “Oh, these other people, they’re bad. I mean they punch holes in the wall and they’ve got baseball bats and I mean they’re REALLY bad, and I’m not like that.” No, no, you just punch me in the head and you know, choke me so that I wonder you know, whether I’m gonna breathe again, but, hey, that's ok because it wasn’t a bat. Gee [laughs], now I can look it with almost humour and realise how ridiculous it is.

Other women described a process of perceived alignment, whereby MBCP group participants were said to have supported their partners’ position of resistance. For example, Paige recalled the following:

He would go in there [the MBCP] and then come home and tell me that the group agreed with him that the kids were at fault. That if the kids wouldn’t do what they did, then he wouldn’t lose his temper and he wouldn't have to hit ‘em — So it was like they were justifying his actions. So he would go to the group and he would justify why he had hit one of the boys and then the men in the group would say, you know, “I would have done that, too, if that was mine” [ ]. And he didn’t learn a damn thing. All he got was that he was in the right, that these people in this group justified the fact that he was right in the actions that he did. And still did.

Based on interpretations made by the women, it appears the group dynamics of the MBCP cultivated an environment where perpetrator resistance could flourish. Paige's narrative above conveys the impact this enduring resistance had on the women. While facing this, the women's initial feelings of hope shifted to frustration, and most women concluded that their partner was not separate from the violence that they perpetrated. Consequently, most of the perpetrators came to be viewed by the women as incapable of change—for example, Janet stated:

I don’t know that you can fix someone like that – I see it that he was thrown a lifeline but he still chose not to accept that help. So I’m just guessing that people like him, unless they get a brain transplant they ain’t gonna change.

Meera was the exception to this, as she blamed her partners violence largely on the influence that his family of origin had:

…it was quite challenging for me going into that family when everybody is trying to control you and, like, main thing was in my in-laws’ family, it was like, [speaking as though the father-in-law] “Oh, woman doesn’t have an opinion. We are the men, we know the best.”—I never questioned his [partner's] responsibility, his love, or, like, his effort to save that marriage. I never questioned that, but it was just he wasn’t able to filter that, whatever was coming through his parents.

#### Assuming and resisting shared responsibility

The referral experience did not involve a linear progression from one emotional or cognitive state to another for the women in this study, most alternated between assuming and resisting shared responsibility. Therefore, this theme demonstrates the interrelated influences of feeling blamed and facing perpetrator resistance. The following extract conveys Fiona’s recollection of when the police were called to her home for the first time. Here you can see the overwhelming “guilt” she experienced:

They [the police] took him and he wasn’t coming back. And then it was just, you feel so guilty – like, what have I done? – Just thinking about “What have I done? What have I done? Like the family's just gone, I’ve just done this” … and my guts were a mess; it was a mess.

In addition to assuming some responsibility as Fiona had, other women like Clara expressed uncertainty as to whether they had, in fact, experienced FV:

I suppose I thought, “Oh well, I had been horrible to him as well.” And that it was more kind of, you know, we were both awful in different ways and I, and I had been awful to him at times you know so it probably didn’t, yeah, yeah, didn’t seem, didn’t feel like it fit the [FV] model that we would hear about, or that you know, was as clear cut.

Shared responsibility flourished in this environment of uncertainty, as the label of victim survivor was one that Clara and a number of others did not appear to immediately associate with.

Regardless of the state they were in (i.e., whether assuming or resisting responsibility), many of the women wanted their partners to be held accountable. As time went on and this did not appear to happen, they developed feelings of disappointment and frustration. For example:

Rose: I went, “Ugh, that's it? Really? That's your punishment? You get to go and sit in a room full of people who don’t think they’ve done anything wrong, and, get to, basically, just nod and smile while you’re there and then leave and be like, “Well, no one's chasing this up.”

Paige: They sit back for eight weeks, or for whatever it is, and at the end of the eight weeks, they walk off! There's no accountability. They go to that group [the MBCP]; they can go home; they can still beat their wives.

Another factor in the women's experience of assuming and resisting responsibility, was the positioning of the FV within family life. For example, Erin stated:

I remember at one stage actually, ah having like a family meeting all of us, and, we looked at what things we thought weren’t good as a family and what things, each of us individually needed to work on to improve the family dynamics so that it wasn't about him.

While assuming responsibility was reinforced by others, over time most women began to question their role in the violence, and eventually, to resist responsibility with confidence. Janet articulated it this way:

He's the sort of person who just always changes the goal posts; it doesn’t matter what you do. I’ve realised now that it wouldn’t matter – I could do what he said every single time and it would always be wrong, so, it's not my, it's not my, burden to bear anymore; that's his.

### Theme 3: “You’ve Gotta Take the Ugly Truth for What It Is”

The third major theme that was revealed through analysis of the women's narratives, speaks to the dynamic nature of concepts related to the visibility of FV that characterized the referral experience. These concepts are presented within the following four sub-themes: (a) receiving external validation; (b) seeing the abuse/r; (c) responding with indignation; and (d) assessing change, assessing the future. The suggestion that “You’ve gotta take the ugly truth for what it is” has been interpreted to mean the women were coming to terms with the reality of their partners’ behaviour and his resistance to change.

#### Receiving external validation

The enhanced visibility of FV that took place during the referral experience validated the women’s assessments regarding how they had been treated and why. For example, Clara recalled the following:

I remember when we were at the “psych” [psychiatric] ward they were like [speaking to partner] “You can’t do that you can’t just smash a window – like that's not okay.” – Like I remember one lady being a bit like, one of the nurses, being a bit like, she just couldn’t believe that that would be something that someone would do, you know like really perplexed and they thought, I think they gave us some money to get them [the windows] fixed. I think that's why it sticks in my head because they were like “Well that's not fair.” You know, like “Here, get the window fixed.”

Clara lived her partners violence every day—it was her “norm”—however, the workers response strongly suggested that it was not ok for her partner to behave in that way. Fiona had a similar experience:

[The women's FV agency] had rang me and they said they wanna talk to me to try and get me out of the house I was in.

This phone call was experienced as validation that her partner was not safe to be around—his behaviour was not ok.

At this point in the referral period, the validation that the women received appears to have been equally, if not more powerful than the blame they experienced. It is possible this was because the messages they received confirmed their own beliefs regarding how family should treat one and other.

#### Seeing the abuse(r)

As a sense of validation permeated the women’s minds, they increasingly saw the abuse as such, and their partners therefore as perpetrators. Erin reflected:

I think when it had escalated to the point where it was the intervention order – the first one – and the referral to the men's behaviour change program, and I think, that even though I was in a state of hope that those things would help, it, in a sense I think it did start me thinking, about things differently, um, cos it was a series of external areas involved and, and I think that does make you reassess things.

Throughout this process the women began to experience a sense of separation between themselves and their partners due to the dissimilarities that they identified. Fiona recalled:

We said we wanted to go to the presentation night, my eldest's presentation night, and, yeah [perpetrator says] “Nope, you’re not going nowhere, youse wanna go there tarting around.” [Fiona] “No, I wanna go and support cos no one's going from the [sporting] house, so we’ll go and show our support for the [sporting] house, so we’ll dress up”…and [perpetrator says] “No, you’re staying home, you’re staying home, you’re fucking staying home. You’re staying home; this is where you belong” — Telling me what I am and what I do and how I don’t respect him, RESPECT him. He's just a prick, a fucking prick of a person [sigh].

While for Fiona it was important to support her child and their sporting house, her partner did not act in a way that suggested he felt the same. When he took this one step further and prevented Fiona from attending, she may have experienced this as a conflict of values or priorities. As a result, this appears to have reinforced a growing sense of separateness.

#### Responding with indignation

As a consequence of the interrelated experiences outlined above, the women began responding to their partners and their situation with increasing indignation. Indignation is a form of anger entwined with an individual’s morals (Jasper, 2014) and is evident within the women’s narratives via their expressions of self-assertion (i.e., the forceful expression of their beliefs and values):

Paige: I said to him, “That's it I've had enough get out, I'm finished that's it, you haven't changed you're just a bully you’re this that the other get out.”

Clara: I said: “You’re saying you’re going to leave me, so if you’re gonna leave pack your bags and leave but otherwise you’re just trying to make me stop telling you how I feel, by telling me you’re gonna leave you know? You’re just trying to control what I’m saying, but making that threat, it's not ok you know? It's just pointless and hurtful.”

Jane: I said: “You’ve hurt a lot of people” and I said: “You’re not taking ownership.”

Erin: I said “No, I’m done with one more shots, I’m done that's it”. Yeah so that was it, it like, it just sort of sucked everything out of me and I thought this is not fun. Not doing this anymore, so that, that was what gave me the strength to say “Go away” but it took a lot of [deep breath] hiccups to get to that point.

Meera recalled her partner returning from an MBCP intake session and expressing his concern about the financial burden that attendance would place on their family. He asserted to Meera that given he was “already sorry,” his attendance at the MBCP would not be beneficial. Meera recalls her response to this as follows:

I was like “But I need something which made me trust.” And then we just had, like, a random discussion. He said: “I can give you a stat dec. [statutory declaration].” So, and I took that in writing that I won’t be living as husband and wife until I feel that things are normal. And if in future for, any of his behaviour I choose to, make any decision like I want to go back to my home country, or if I want to, you know, just get IVO back again he wouldn’t stop me, and he did that — But I was like so firm this is what I want and I told to my husband: “I know we shouldn’t be having any condition in our marriage, but you don’t left me with any other choice, and for me this is the last effort just to save everything, and if it doesn’t work, it doesn’t work, we move on.”

During the referral period Angeline's partner was physically violent towards her and then instantly denied it. When her partner later stated that he would never assault her again, she responded with: “Well if you ever do that again it will be over, you can’t ever touch me like that again.”

Janet asserted herself and her right to safety by reporting her partner's breaches:

Two days after we had that family report done, he breached the IVO and, um, I actually went and made a statement this time [crying], the other times that he’d breached I didn’t because I felt that I would be being just a nuisance, but every time I’ve spoken to the police they’ve been awesome and really helpful and I haven’t had anyone that's been horrible; been really blessed that every single person that I’ve dealt with has been fabulous.

Rose, on the other hand, had not found her experience with police to be supportive: “Oh, they made me feel like shit. They were very victim blaming. “Well, why didn’t you come sooner?” [police—patronising tone]. In Rose's case, it would appear that her indignation was prompted by what she perceived as an unfair response from the police and other service providers.

The increasing sense of indignation experienced by the women consolidated their view that their partner had been responsible for his own behaviour and that it was he that needed to change.

#### Assessing change, assessing the future

Through analysis of the women’s narratives, the experience of referral was shown to involve a process of pivotal assessment for victim survivors. Effectively, it created a new environment for the women to assess their situation and their partners’ behavior. Both Meera and Angeline identified some change in their partners - for example, Meera stated:

Because I feel when. he got that call for referral and he's been there, I feel maybe he, he realised at that stage how serious it was, maybe I feel — Maybe it gave him a chance to see, where he is standing at this stage, although he hadn’t been to the program but just, going through that intake program. Yeah so it has played a vital role, but, because he hadn’t actually attended it was just the referral.

The other women in this study however, determined that change was unlikely to ever take place.

## Discussion

In addition to the major themes and sub-themes that arose through analysis of the women's narratives, several significant insights were formulated through a process of relating the research findings to existing literature and relevant theoretical constructs. A brief summation of these appears here, along with reflection on the potential implications for FV practice and reform.

### Insight 1: (Baseless) Hope

The experiences shared by the women in this study around their initial state of hopefulness, suggests that the attention MBCPs have received over the years has generated confidence within the community concerning their ability to affect meaningful change. As a result, the women's hopes of change were heightened when the referral was made. However, the lived experience that eventuated was characterized by a considerable lack of support for the women. Combined with the consistently unverifiable outcomes of MBCPs (McGinn et al., 2021), it appears their hopes were baseless. This brings into question the ethics of raising expectations when the efficacy of MBCPs is still widely contested and when only minimal support is typically offered to women during the referral period (Smith et al., 2013). Furthermore, it appears that greater consideration regarding the impact of an MBCP referral on victim survivors, is necessary (Zeuschner, 2022).

### Insight 2: Contradictory Expectations

Exploration of the women's lived experiences reveals the complexity of the victim blaming that took place. The women were regularly confronted with contradictory expectations around protecting themselves and their children by leaving their violent partners (or reaching out for support), while also bearing sole responsibility for maintaining the family structure by staying with their violent partners and “keeping the peace”. This incongruence created a strong sense of dissonance for each of them, and we can see that as the women grappled with the competing pressures, they were in effect working towards reducing the discomfort it produced. The outcome was a tumultuous period where they all fluctuated between assuming and resisting responsibility for the violence perpetrated against them. Over time, the women increasingly, and notably, identified the dissimilarities between themselves and their partners—largely due to their partners’ unwillingness to take responsibility and change for the family. The opposing, and at times invalidating messages that the women received throughout the referral period suggests that significant effort is still required to change community perceptions and improve FV service response (Zeuschner, 2022).

### Insight 3: Pivotal Assessment

It appears that MBCP referrals can generate a critical period in time for victim survivors, by creating an expectation of change. As evidenced by the experiences of the women in this study, when an individual perceives an injustice to have occurred, a moral to have been crossed, or if their sense of self is threatened, a powerful felt experience of indignation can follow. Their sense of indignation appeared to be instrumental in assisting them to confidently formulate their own pivotal assessment of their partners’ capacity and/or willingness to change. In essence, it helped them to believe with confidence that their partners’ behaviour was unacceptable and unwarranted; that he was responsible for his own behaviour and that it was he who needed to change.

Despite contrasting with the many messages they received from others, including family members, friends, and service providers, the sense of indignation that each woman experienced energized their activism—action that led to life-altering changes for each of them. This study stresses the importance of supporting victim survivors to identify, connect with, and gain confidence in acknowledging their own realities and beliefs regarding their partners’ behaviour. Furthermore, it emphasizes the significance of supporting victim survivors to identify who is responsible for that behaviour (Zeuschner, 2022).

## Conclusion

Referring male perpetrators of violence to MBCPs triggers a period of pivotal assessment for victim survivors and should be recognized as a significant event in and of itself. Even if the male perpetrator being referred does not go on to engage with an MBCP, it is clear the suggestion that he needs to change still plays an important role due to the meaning the women ascribe to their experiences of this. The women whose insights have been shared, were initially deeply invested in the hope of change referral fueled. As a result, they were deeply affected by their partners’ responses, as well as by the responses they received from family, friends, and service providers. Therefore, while for workers referral may be viewed as an administrative process or weighted suggestion, for victim survivors it must be recognized as signifying far more. The needs of victim survivors for support and safety during referral, are integral to the change that is being sought and pivotal to the decisions of women about the future of their relationships. While the experiences of the women who participated in this study cannot be generalized to all women, their stories highlight the centrality of victim survivors to the effects and implications of an MBCP referral. As such, their experiences and insights must inform the delivery of community services—including administrative processes such as referral.

## References

[bibr1-10778012251338487] Alase A. (2017). The interpretative phenomenological analysis (IPA): A guide to a good qualitative research approach. International Journal of Education and Literacy Studies, 5(2), 9–19. 10.7575/aiac.ijels.v.5n.2p.9

[bibr2-10778012251338487] Alexander P. Morris E. Tracy A. Frye A. (2010). Stages of change and the group treatment of batterers: A randomized clinical trial. Violence & Victims, 25(5), 571–587. 10.1891/0886-6708.25.5.571 21061865

[bibr3-10778012251338487] Australian Institute of Health and Welfare. (2024). Family, domestic and sexual violence: Summary. https://www.aihw.gov.au/family-domestic-and-sexual-violence/resources/fdsv-summary

[bibr4-10778012251338487] Bowden P. Mummery J. (2009). Understanding feminism. Acumen.

[bibr5-10778012251338487] Burgess-Proctor A. (2015). Methodological and ethical issues in feminist research with abused women: Reflections on participants’ vulnerability and empowerment. Women’s Studies International Forum, 48, 124–134. 10.1016/j.wsif.2014.10.014

[bibr6-10778012251338487] Bynum W. Varpio L. (2018). When I say … hermeneutic phenomenology. Medical Education, 52(3), 252–253. 10.1111/medu.13414 28895184

[bibr7-10778012251338487] Chung D. Upton-Davis K. Cordier R. Campbell E. Wong T. Salter M. Austen S. O’Leary P. Breckenridge J. Vlais R. Green D. Pracilio A. Young A. Gore A. Watts L. Wilkes-Gillan S. Speyer R. Mahoney N. Anderson S. Bissett T. (2020). *Improved accountability: The role of perpetrator intervention systems* (Research Report Issue 20 – June 2020). Australia’s National Research Organisation for Women’s Safety. https://www.anrows.org.au/publication/improved-accountability-the-role-of-perpetrator-intervention-systems/

[bibr8-10778012251338487] Commonwealth of Australia. (2022). The National Plan to End Violence Against Women 2022-2032. Commonwealth Government of Australia, Department of Social Services.

[bibr9-10778012251338487] Eckhardt C. Holtzworth-Munroe A. Norlander B. Sibley A. Cahill M. (2008). Readiness to change, partner violence subtypes, and treatment outcomes among men in treatment for partner assault. Violence & Victims, 23(4), 446–475. 10.1891/0886-6708.23.4.446 18788338

[bibr10-10778012251338487] Gray R. Broady T. Gaffney I. Lewis P. Mokany T. O'Neill B. (2016). I’m working towards getting back together: Client accounts of motivation related to relationship status in men’s behaviour change programmes in New South Wales. Australia. Child Abuse Review, 25(3), 171–182. 10.1002/car.2318

[bibr11-10778012251338487] Gringeri C. E. Wahab S. Anderson-Nathe B. (2010). What makes it feminist? Mapping the landscape of feminist social work research. Affilia, 25(4), 390–405. 10.1177/0886109910384072

[bibr12-10778012251338487] Grosser K. Tyler M. (2022). Sexual harassment, sexual violence and CSR: Radical feminist theory and a human rights perspective. Journal of Business Ethics, 177(2), 217–232. 10.1007/s10551-020-04724-w

[bibr13-10778012251338487] Hossain M. Zimmerman C. Kiss L. Abramsky T. Kone D. Bakayoko-Topolska M. Annan J. Lehmann H. Watts C. (2014). Working with men to prevent intimate partner violence in a conflict-affected setting: A pilot cluster randomized controlled trial in rural Côte d’Ivoire. BMC Public Health, 14(339). 10.1186/1471-2458-14-339 PMC4007144 24716478

[bibr14-10778012251338487] Jasper J. M. (2014). Constructing indignation: Anger dynamics in protest movements. Emotion Review, 6(3), 208–213. 10.1177/1754073914522863

[bibr15-10778012251338487] Kaboub F. (2008). Positivist paradigm. Encyclopaedia of Counselling, 2, 343. http://personal.denison.edu/~kaboubf/Pub/2008-Positivist-Paradigm.pdf

[bibr16-10778012251338487] McConnell N. Taylor J. (2016). Evaluating programmes for violent fathers: Challenges and ethical review. Child Abuse Review, 25(3), 183–191. 10.1002/car.2342

[bibr17-10778012251338487] McGinn T. Taylor B. McColgan M. (2021). A qualitative study of the perspectives of domestic violence survivors on behavior change programs with perpetrators. Journal of Interpersonal Violence, 36(17-18), NP9364–NP9390. 10.1177/0886260519855663 31216924

[bibr18-10778012251338487] Milner J. Singleton T. (2008). Domestic violence: Solution-focused practice with men and women who are violent. Journal of Family Therapy, 30(1), 29–53. 10.1111/j.1467-6427.2008.00414.x

[bibr19-10778012251338487] Morran D. (2013). Desisting from domestic abuse: Influences, patterns and processes in the lives of formerly abusive men. Howard Journal of Criminal Justice, 52(3), 306–320. 10.1111/hojo.12016

[bibr20-10778012251338487] Musser P. H. Semiatin J. N. Taft C. T. Murphy C. M. (2008). Motivational interviewing as a pregroup intervention for partner-violent men. Violence & Victims, 23(5), 539–557. 10.1891/0886-6708.23.5.539 18958984

[bibr21-10778012251338487] Richards J. C. MacLachlan A. J. Scott W. Gregory R. (2004). Understanding male domestic partner abusers. Trends & Issues in Crime & Criminal Justice, 283, 1–6. https://www.aic.gov.au/sites/default/files/2020-05/tandi283.pdf

[bibr22-10778012251338487] Royal Commission into Family Violence (RCFV). (2016). *Summary and recommendations* [Parl Paper No 132 (2014–16)]. http://rcfv.archive.royalcommission.vic.gov.au/MediaLibraries/RCFamilyViolence/Reports/RCFV_Full_Report_Interactive.pdf

[bibr23-10778012251338487] Simmons C. Lehmann P. Collier-Tenison S. (2008). Men’s use of controlling behaviors: A comparison of reports by women in a domestic violence shelter and women in a domestic violence offender program. Journal of Family Violence, 23(6), 387–394. 10.1007/s10896-008-9159-6

[bibr24-10778012251338487] Smith J. A. Flowers P. Larkin M. (2009). Interpretative phenomenological analysis: Theory, method and research. Sage Publishing Ltd.

[bibr25-10778012251338487] Smith J. Humphreys C. Laming C. (2013). The central place of women’s support and partner contact in men’s behaviour change programs. Ending Men’s Violence Against Women and Children, 1, 7–28. https://www.researchgate.net/publication/297295702_The_central_place_of_women's_support_and_partner_contact_in_men's_behaviour_change_programs

[bibr26-10778012251338487] Smith J. A. Nizza I. E. (2021). Essentials of interpretative phenomenological analysis. American Psychological Association.

[bibr27-10778012251338487] Stanley N. Graham-Kevan N. Borthwick R. (2012). Fathers and domestic violence: Building motivation for change through perpetrator programmes. Child Abuse Review, 21(4), 264–274. 10.1002/car.2222

[bibr28-10778012251338487] State Government of Victoria. (2021). What is family violence? https://www.vic.gov.au/what-family-violence

[bibr29-10778012251338487] van Manen M. (2016). Researching lived experience: Human science for an action sensitive pedagogy. Routledge.

[bibr30-10778012251338487] Women with Disabilities Victoria. (2023). Taking action guide: Prevention of violence against women with disabilities. https://www.wdv.org.au/family-violence-resources/

[bibr31-10778012251338487] Zeuschner L. (2022). Women’s lived experiences of their partners’ referral to a men’s behaviour change program: A feminist interpretative phenomenological analysis [Doctoral dissertation], Federation University Australia https://primoapac01.hosted.exlibrisgroup.com/permalink/f/13o3vsv/UB_VITAL16430

